# Serological survey on Hepatitis E virus in Namibian dogs, cats, horses, and donkeys

**DOI:** 10.3389/fvets.2024.1422001

**Published:** 2024-07-18

**Authors:** Umberto Molini, Giovanni Franzo, Lourens de Villiers, Leandra van Zyl, Mari de Villiers, Siegfried Khaiseb, Frank Busch, Sascha Knauf, Klaas Dietze, Martin Eiden

**Affiliations:** ^1^School of Veterinary Medicine, Faculty of Health Sciences and Veterinary Medicine, University of Namibia, Windhoek, Namibia; ^2^Central Veterinary Laboratory (CVL), Windhoek, Namibia; ^3^Department of Animal Medicine, Production and Health, University of Padova, Legnaro, Italy; ^4^Institute of International Animal Health/One Health, Friedrich-Loeffler-Institut, Federal Institute for Animal Health, Greifswald – Insel Riems, Germany; ^5^One Health/International Animal Health, Faculty of Veterinary Medicine, Justus Liebig University, Giessen, Germany; ^6^Institute for Novel and Emerging Infectious Diseases, Friedrich-Loeffler-Institut, Greifswald-Insel Riems, Germany

**Keywords:** HEV, Namibia, seropositivity, pets, horses, donkeys, zoonosis

## Abstract

The present study investigated the seropositivity rate of Hepatitis E virus (HEV) in domestic and working animals in Namibia, which included dogs, cats, horses, and donkeys. HEV poses a growing threat as a significant cause of human hepatitis globally and has several genotypes of varying zoonotic potential. As epidemiological data on the seroprevalence of HEV in Namibia is scarce, a serosurvey was conducted on archived serum samples of 374 dogs, 238 cats, 98 horses, and 60 donkeys collected between 2018 and 2022 from different regions, to assess the potential of these animals as sources of HEV infection. The findings revealed that 10.43% (*n* = 39/374) canine and 5.88% (*n* = 14/238) feline samples tested positive for HEV antibodies, whereas no seropositivity was detected in horses and donkeys. The study further examined the risk factors associated with HEV seropositivity, including animal sex, age, and geographical region, and noted a higher prevalence in dogs living in areas with intensive pig farming. Although there is no direct evidence indicating that these animals served as major reservoirs for HEV transmission to humans, the study underscores the importance of preventive measures to minimize contact exposure with pets considering the potential zoonotic risk, especially for susceptible risk groups. Further research is needed to explore the zoonotic potential of domestic animals and the epidemiological links between animal and human HEV transmissions in Namibia.

## 1 Introduction

The Hepatitis E virus (HEV) is increasingly recognized worldwide as a significant cause of hepatitis in humans. The World Health Organization (WHO) estimates 20 million global HEV infections annually (1). The disease’s generally low severity and fatality rate, ranging from 0.2 to 4.0%, can escalate to 30.0% in pregnant women (2–4). Moreover, more severe and chronic forms of hepatitis are able to develop in immunocompromised patients (5). An estimated 20.0 million cases of acute Hepatitis E in 2019 and approximately 44,000 deaths were reported representing 3.3% of all viral hepatitis-related mortality (6).

HEV is a non-segmented, quasi-enveloped, single-stranded RNA virus classified into the *Hepeviridae* family (7). The most relevant group for mammals belongs to the genus *Paslahepevirus*, species *Paslahepevirus balyani*^1^ and eight genotypes have been identified to date. Genotypes 1 and 2 (HEV-1/−2) are known to infect only humans and have been responsible for significant outbreaks in developing countries due to contamination of drinking water and food with human waste in areas with poor hygiene practices (8).

In contrast, genotypes 3 and 4 are considered true zoonotic genotypes (9). Main reservoirs for HEV-3 are domestic pigs (*Sus scrofa domesticus*) and wild boar (*Sus scrofa*), with additional involvement from wild animals, such as rabbits (*Oryctolagus cuniculus*) (10–12), and deer species including roe deer (*Capreolus capreolus*), fallow deer (*Dama dama*) and red deer (*Cervus elaphus*) (13–15).

Similarly, HEV-4’s primary reservoir hosts are domestic pigs, but infections have been documented also in wild boar, deer, cattle, goat, sheep, donkeys (16). Genotypes 5 and 6 have been identified only in Japanese wild boars (17). HEV-7 and HEV-8 have been found in dromedary *(Camelus dromedaries)* and Bactrian camels *(Camelus bactrianus),* respectively. One case of HEV-7 infection was reported in an immunocompromised patient who consumed camel milk and meat, suggesting a zoonotic potential (18).

HEV-3 and HEV-4 are the main sources of zoonotic HEV infection in humans: This includes ingestion of raw and undercooked meat products (19), as well as direct transmission by direct and work-related contact with infected animals and the contamination of water sources (19). This leads to higher antibody prevalence among individuals in contact with domestic and wild pigs, such as slaughterhouse workers, farmers, veterinarians, and hunters (20, 21). Additional cases of acute and chronic infections originating from rabbit HEV have been reported in patients from France and Switzerland (10, 22). Finally, an increasing number of human hepatitis caused by rat-derived HEV from the genus *Rocahepevirus* (species *Orthohepevirus ratti*) are currently being observed (23) highlighting the general zoonotic risk associated with hepevirus. The wide and continuously expanding host range of HEV, and considerations toward its direct transmission from animal reservoirs, prompts further investigation into the role of companion and working animals as potential sources of infection. Serological evidence of HEV infection in dogs, cats and horses illustrates this aspect (9, 24, 25). Viral presence of HEV has been confirmed through molecular assays in horses (26). While these animals are unlikely to serve as major host reservoirs, considerations toward the close contact humans have with potential sources of HEV infection are warranted, in particular for known susceptible risk groups such as immunocompromised individuals. No epidemiological evidence of HEV circulation in companion animals has previously been reported in Namibia nor from other countries in Sub-Saharan Africa. Considering this lack of information and the potential public health implications, in this study a serosurvey was conducted on archived samples from dogs, cats, horses, and donkeys, providing the first insight into HEV seropositivity in the country.

## 2 Materials and methods

### 2.1 Sample size, origin, and patient signalment

**Figure 1:**
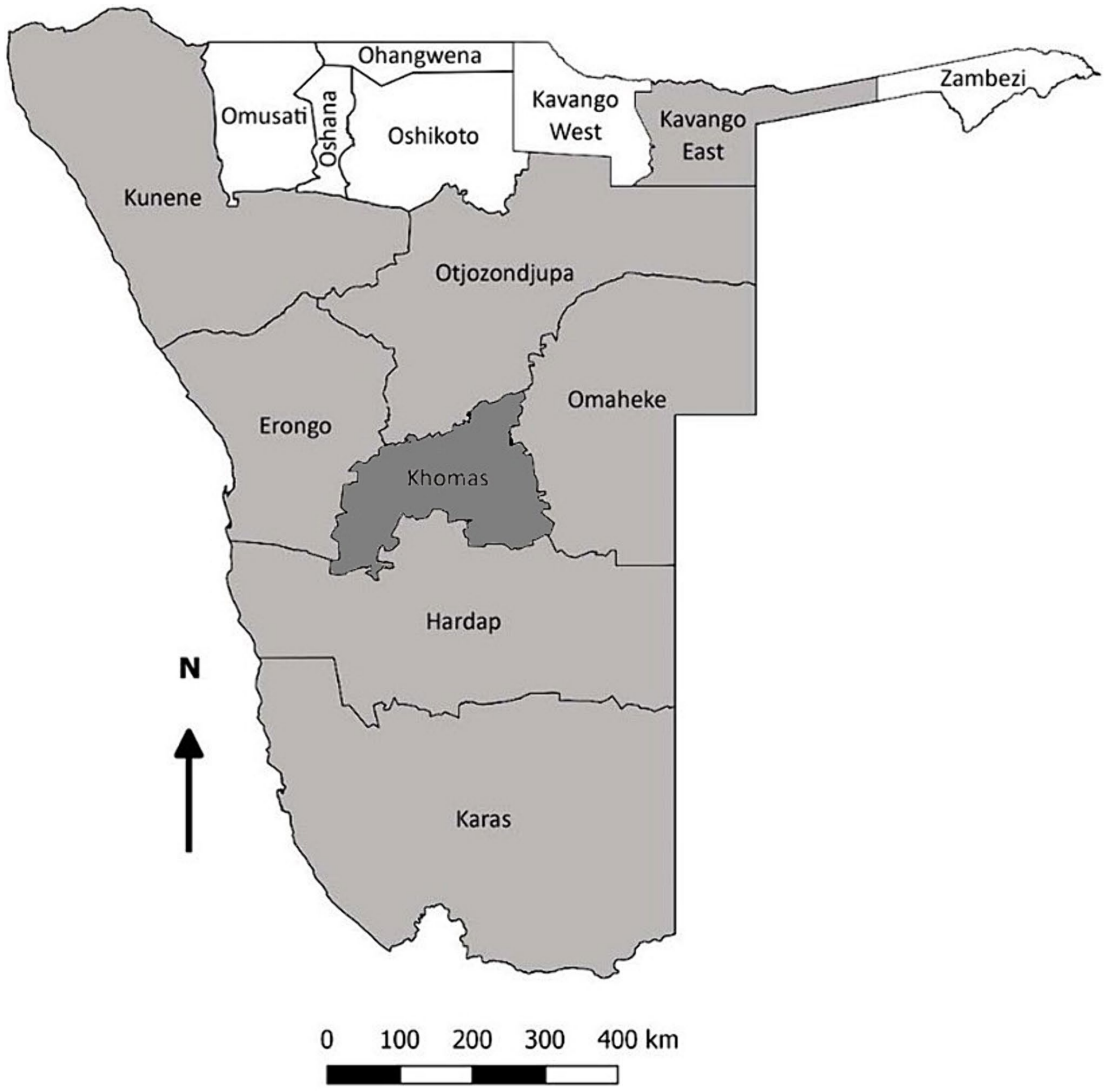
Map of Namibia demonstrating the regions of sample origin (shaded). The Khomas region, where also donkeys and horses were sampled, is highlighted in dark gray.

Archived serum samples were evaluated, collected between October 2018 to September 2022, from domestic dogs, cats, and equids during routine veterinary services provided by the School of Veterinary Medicine, University of Namibia. As seen in Figure 1, samples originated from across eight regions of Namibia, namely Omaheke, Erongo, Khomas, Otjozondjupa, Kunene, Kavango-East, Hardap, and Karas. Available metadata on patient signalment were reported, including patient sex, breed, and age.

#### 2.1.1 Companion animals

The canine and feline samples originated from several regions of Namibia. A total of 374 dog and 238 cat archived samples were available for analysis in this study. Based on the available sample size, the infection presence at the population level could be demonstrated with a sensitivity of 95% if the population prevalence was, respectively, at least 0.8 and 1.3%, assuming a test sensitivity of 95%.^2^ Signalment of the 374 dog samples studied included 173 females and 201 males; 339 crossbreeds and 35 purebreds; as well as 98 dogs less than 1 year of age, and 276 older than 1 year. In contrast, signalment of the 238 cat samples included 118 females and 120 males; all were mixed-breed, 74 were younger than 1 year, and 164 cats older than 1 year of age.

#### 2.1.2 Horses

The analyzed archived equine serum samples were collected from 98 healthy horses located at three different stables in the Windhoek city area. Four of the 98 horses had been imported from South Africa, while the others were born and raised in Namibia and had not traveled outside the Windhoek district at the time of sampling. The infection presence at the population level could be demonstrated with a sensitivity of 95% if the population prevalence was at least 3.2%, assuming a test sensitivity of 95%. The signalment of the equine cohort included 64 geldings, 33 mares, and one stallion. The breed composition predominately included 56 Warmbloods, 16 Arabians, 9 Quarter horses, and the remaining 17 horses belonged to other breeds. The horses were aged between 2 and 27 years of age.

#### 2.1.3 Donkeys

One hundred and sixty archived donkey samples were analyzed, which were randomly collected from healthy donkeys from several regions of Namibia. None of the donkeys had a history of leaving their region of origin, or of having traveled between different regions. The infection presence at the population level could be demonstrated with a sensitivity of 95% if the population prevalence was at least 5.1%, assuming a test sensitivity of 95%. The signalment of the donkey cohort included 68 males and 92 females; no specific metadata on donkey breed; and animals aged between 3 and 5 years.

### 2.2 Sample processing

Archived serum aliquots, stored at −20°C, were thawed and underwent centrifugation at 2500 g for 10 min before analysis. All species-specific serum aliquots were screened for HEV antibodies using a commercial double-antigen multi-species sandwich ELISA (HEV ELISA 4.0v, MP Biomedicals Germany GmbH, Eschwege). Optical density (OD) was read at 450 nm using a single filter plate reader (Thermo Scientific Multiskan EX, Waltham, Massachusetts, United States) as instructed by the manufacturer. The ELISA kit has a sensitivity and specificity of 99.2% and has been proven to detect anti-HEV antibodies in serum or plasma in a wide range of animal species, including domestic and wild cats, dogs, pigs and horses (25, 27, 28).

### 2.3 Statistical analysis

The proportion of positive samples was calculated for each species, and the 95% confidence interval (CI) was estimated using a normal approximation to the binomial calculation. The odds ratios of seropositivity were calculated for different sample features, including sample origin and associated patient signalment, by fitting logistic regression models. To assess the potential impact of living in areas featured by high-density pig farming, regions were dichotomized and aggregated into two categories, namely “high” and “low”-density pig farming. The significance level was set at *p* < 0.05. All analyses were performed using R software, version 4.2.2 (29).

## 3 Results

### 3.1 Seroprevalence and risk

Thirty-nine out of 374 canine samples (10.43%, CI 7.33–13.53%) and 14 out of 238 feline samples (5.88%, CI 2.89–8.87%) tested seropositive for HEV on ELISA, as shown in Table 1. No tested horses and donkey samples showed seroconversion for HEV.

**Table 1:** Seroprevalence of HEV in domestic and working animals across several regions of Namibia.

Region	N. dogs	cELISA + (%)	N. cats	cELISA + (%)	N. horses	cELISA + (%)	N. donkeys	cELISA + (%)
Omaheke	47	0 (0.0%)	28	0 (0.0%)	0	0 (0.0%)	20	0 (0.0%)
Erongo	47	3 (6.38%)	41	1 (2.44%)	0	0 (0.0%)	20	0 (0.0%)
Khomas	50	3 (6.00%)	51	0 (0.0%)	98	0 (0.0%)	20	0 (0.0%)
Otjozondjupa	47	14 (29.79%)	11	1 (9.09%)	0	0 (0.0%)	20	0 (0.0%)
Kunene	47	2 (4.25%)	8	0 (0.0%)	0	0 (0.0%)	20	0 (0.0%)
Kavango East	47	8 (17.02%)	39	6 (15.38%)	0	0 (0.0%)	20	0 (0.0%)
Karas	42	6 (12.76%)	26	0 (0.0%)	0	0 (0.0%)	20	0 (0.0%)
Hardap	47	3 (6.38%)	34	6 (17.65%)	0	0 (0.0%)	20	0 (0.0%)
TOTAL	374	39 (10.43%)	238	14 (5.88%)	98	0 (0.0%)	160	0 (0.0%)

The odds were 2.51 times higher (CI 1.28–4.97; *p* = 0.001) in regions featured by intensive pig farming. No significant gender, age or breed effect was observed in dogs. Seropositivity in cats was not affected by any of the population features considered.

### 3.2 Clinical features in seropositive patients

Overall, 35/39 (89.74%) of HEV positive dogs were associated with metadata reflecting at least one of the following clinical signs: neurological, respiratory, enteric, or systemic (pyrexia, weight loss, abdominal enlargement, and/or lymphadenopathy). Positive samples were associated with the following signs: 0/39 (0%) neurological; 7/39 (17.95%) respiratory; 3/39 (7.69%) enteric; 6/39 (15.38%) pyrexia; 8/39 (20.51%) weight loss; 14/39 (35.9%) abdominal enlargement; and 14/39 (35.9%) lymphadenopathy, respectively (Table 2). Concerning the clinical signs found in cats, 9/14 (64.29%) of HEV positive felines were associated with metadata reflecting at least one of the clinical symptoms indicated: 0/14 (0%) neurological; 3/14 (21.43%) respiratory; 0/14 (0%) enteric; 2/14 (14.29%) pyrexia; 1/14 (7.14%) weight loss; 2/14 (14.29%) abdominal enlargement; and 3/14 (21.43%) lymphadenopathy, respectively (Table 2). None of the equid patients had metadata associated with abnormal clinical features at the time of sample collection and were seemingly healthy at presentation. A complete list of clinical records associated with the considered cases is reported in Supplementary Table S1.

**Table 2:** Clinical features in dogs and cats tested positive for HEV.

Clinical features in positive animals	N. dogs +	(%)	N. cats +	(%)
Neurological	0/39	(0.0%)	0/14	(0.0%)
Respiratory	7/39	(17.95%)	3/14	(21.43%)
Enteric	3/39	(7.69%)	0/14	(0.0%)
Pyrexia	6/39	(15.38%)	2/14	(14.29%)
Weight loss	8/39	(20.51%)	1/14	(7.14%)
Abdominal enlargement	14/39	(35.9%)	2/14	(14.29%)
Lymphadenopathy	14/39	(35.9%)	3/14	(21.43%)
TOTAL	35/39	(89.74%)	9/14	(64.29%)

Summary table reporting the number and percentage of positive dogs and cats showing clinical signs.

## 4 Discussion

HEV represents a threat to human health, with several outbreaks recorded worldwide, especially in Sub-Saharan Africa (30–32). Namibia suffered from several HEV outbreaks starting from a 1983 outbreak in Kavango region (33) till a large outbreak from 2017 to 2020 with more than 7,000 human HEV cases in all 14 regions (34). During these outbreaks, genotypes 1 and 2 were identified (33, 35). Both genotypes are exclusively human-associated and are increasingly recognized for their significant role in outbreaks especially in areas with limited access to water and inadequate sanitary conditions and untreated sewage (36). Current epidemiological data regarding zoonotic transmission, and the risks associated with contact exposure to domestic and farm animals are not available from Namibia which would be essential to understand and mitigate potential health threats. For this reason, investigating the occurrence and relative risk factors in these animal populations including companion animals is relevant.

In the present study, 10.43% of archived canine samples from predominately low-income areas and settlements in Namibia were seropositive for HEV antibodies. The observed seropositivity rate is consistent with previous studies on dogs from Germany (10%) (25), Italy (8.2%) (37), Spain (10%) (27), and China (12%) (38). However, results from other studies varied significantly, depending on the population, geographical region, and used test kits, ranging from 0% in Japan (39) to up to 57% in Germany (40). The differences observed among the surveys, aside from being attributable to real differences in the epidemiological scenario, can also be linked to the study design, the population considered, and the tests applied. These limitations, combined with the convenience nature of the sampling, also affected this study and may have at least partially influenced the results.

Differences in exposure risk to infected and/or carrier animals could justify the significant effect of sampling regions, with regions that have poorly developed farming systems or an industrial economy, like Erongo, being at a lower risk of HEV exposure. Conversely, regions with a higher density of pig farms, the main reservoir of HEV-3 and HEV-4, showed higher odds of seropositivity. High contact opportunities with live animals or contaminated fomites might play a role in exposure risk. Although, direct dog-to-pig contact is an unlikely source of infection given commercial pig farms’ biosecurity protocols, waste products, including uncooked bones, offal, and slaughtered pig meat, are commonly used for dog food and nutrition, and may represent a likely route of infection, as previously suggested for other pathogens (41). Nevertheless, the HEV seropositivity rate reported here is comparable to those observed in European countries, where feeding dogs with by-products is less common (27, 42). Therefore, further studies should be conducted to explore this hypothesis, although the increasing popularity of the BARF (Biologically Appropriate Raw Food) diet in high-income countries could help to justify similar scenarios (43). Due to shared environments and facilities with people working in the swine industry, reverse zoonosis events cannot be excluded.

The HEV seropositivity rate in Namibian cats was slightly lower at 5.88%, compared to dogs. The reduced prevalence compared to dogs was also observed in similar studies from Germany (25), Spain (27), and the Netherlands (44). While feeding practices for cats in poor, rural settings are likely similar to those of dogs, cats display significantly different behavior to livestock compared to dogs, and therefore direct contact opportunities with pigs could, in general, be lower. Based on the observed pattern, exposure to HEV reservoirs should be considered, for example contaminated water sources, prey, and other wild animals (45). No horses or donkeys were tested seropositive in this investigation, similar to a horse survey from Korea (46) but in contrast to other studies from Europe (47). Based on the results of this study, the risk of infection in equids can be considered negligible. This could be because they do not live in the same household as their owners, as well as general differences in the handling, transportation, storage and disposal of manure.

In summary, this study highlights a potential risk of HEV exposure by pets in Namibia. This finding could prove especially relevant to risk and vulnerable groups at increased risk of infection, including pregnant women and immunocompromised individuals. Appropriate hygiene measures should be encouraged in routine contact with pets. Companion animal owners should limit their pet’s contact exposure to other potentially infected animals or fomites. Given the known risks to human and animal health, the use of raw pork as dog/cat food in particular should be discouraged. Further investigations into HEV should account for associations with Namibia’s wildlife by assessing the prevalence and incidence of animal infections. HEV exposure has been commonly reported in several wild carnivores and, differently from companion animals, molecular evidence of viral presence was also provided (e.g., in lynx, wolf, and fox) (28, 37, 40, 45, 48, 49). The potential health impacts “of and on” endangered species, the viral exchange with domestic animals, and the epidemiological significance as a source of human infection should be further investigated with a specific focus on Namibia and African countries, also by applying molecular assays. Evaluation of the viral presence, infection duration and shedding level should be further investigated to effectively assess the threat represented by companion animals. Furthermore, there is a need for a better understanding of the extent of the zoonotic potential of HEV-infected domestic animals, especially, but not limited to pigs, of the viral lifecycle and pathogenesis in these hosts, and characterization of circulating HEV strains in Namibia. While the occurrence of productive infections in domestic canids and their infectiveness is still unknown (38, 48), the use of companion animals as sentinels for the assessment of zoonotic disease risks can help determine the direction of HEV contact exposure and clarify the epidemiological links to zoonotic disease infection within the Namibian context. This study provides the first evidence of HEV exposure in companion animals in Namibia, highlighting the need to incorporate a One Health approach when addressing human cases of hepatitis.

## Data availability statement

The original contributions presented in the study are included in the article/Supplementary material, further inquiries can be directed to the corresponding author.

## Ethics statement

Ethical approval was not required for the studies involving animals in accordance with the local legislation and institutional requirements because sampled material originated from the archived sample biobanks. Written informed consent was obtained from the owners for the participation of their animals in this study because samples were collected during routine diagnostic procedures.

## Author contributions

UM: Conceptualization, Funding acquisition, Project administration, Supervision, Writing – original draft. GF: Data curation, Formal analysis, Writing – original draft, Writing – review & editing. LV: Data curation, Formal analysis, Writing – review & editing. LZ: Data curation, Formal analysis, Writing – review & editing. MV: Data curation, Formal analysis, Writing – review & editing. SKh: Data curation, Formal analysis, Writing – review & editing. FB: Data curation, Formal analysis, Project administration, Writing – review & editing. SKn: Formal analysis, Writing – review & editing. KD: Data curation, Formal analysis, Funding acquisition, Project administration, Writing – review & editing. ME: Supervision, Formal analysis, Writing – review & editing.

## Footnotes

^1^ https://ictv.global/taxonomy

^2^ https://epitools.ausvet.com.au

## Supplementary material

Supplementary Table S1 Reporting the metadata and clinical features of the subjects included in the study.
